# Supplementary material for: Tomographic imaging of the photonic environment of plasmonic nanoparticles
Source: Nat Commun. 2017 Jun 26;8:37. doi: 10.1038/s41467-017-00051-3 (PMC5484695; doi:10.1038/s41467-017-00051-3)
Supplement: Supplementary file 1 — Supplementary Information [file 41467_2017_51_MOESM1_ESM.pdf]

File Name: Supplementary Information
Description: Supplementary Figures, Supplementary Notes and Supplementary References

File Name: Supplementary Movie 1
Description: Reconstructed photonic LDOS for nanocuboid. Video showing reconstructed and simulated photonic LDOS for the dipole and quadrupolar mode of the nanocuboid. The colour of the pencils represents the LDOS magnitude, the orientation indicates the direction along which the projected photonic LDOS is maximal. For comparison see also Figs. 2 and 3 of the main manuscript.

File Name: Supplementary Movie 2
Description: Reconstructed LDOS for coupled disks. Video showing reconstructed photonic LDOS for the five main peaks of the coupled nanodisks. The colour of the pencils represents the LDOS magnitude, the orientation indicates the direction along which the projected photonic LDOS is maximal. For comparison see also Figs. 2 and 4 of the main manuscript.

File Name: Peer Review File
Description:

Supplementary Note 1. Contamination during tilt series acquisition

During acquisition of the tilt series, carbon contamination slowly built up on the samples due to irradiation by the electron beam, causing continuous red-shifts of the resonance energies, in agreement to previous experimental studies [1, 2]. Supplementary Fig. 5 shows HAADF STEM images before and after tilt series acquisition highlighting the carbon contamination. Supplementary Fig. 6 shows spectra summed over the full spectrum image (SI) for the nanocuboid sample and for the coupled disks. For each SI the plasmon resonance maps were extracted around the current peak position, i.e. the integration windows were shifted to lower energies for SIs acquired later in the series. While the resonance energies shifted, the modal distribution in the plasmon resonance maps did not change significantly with sample contamination. For each mode resonance maps extracted before, during and after tilt series acquisition are compared in Supplementary Fig. 7. The strongest effect is visible for Mode 1 in the coupled disk sample. Here the resonance shifts to very low energies, too close to the zero-loss peak to allow extraction of the mode for SIs acquired later in the series. For this reason only the first part of the images was used for reconstruction of this mode.

Supplementary Note 2. Simulation and reprojection of tilted EELS maps

Tilted EELS maps were simulated for the cuboid structure without substrate, see Supplementary Fig. 3. The maps show good agreement with experimental maps. Reprojected EEL maps are shown for dipolar and quadrupolar mode of cuboid structure (Supplementary Fig. 3) and mode 1-5 of coupled disks (Supplementary Fig. 4). The reprojections are in good agreement with experimental data set.

Supplementary Note 3. Estimation of projection quality

The signal to noise ratio (SNR) in the projections can be calculated in analogy to the scheme proposed by Egerton in [3], Eq. (5.23). Assuming Poissonian counting statistics and detector noise the uncertainty associated with the extraction of a weak signal is determined by

$$SNR = DQE^{1/2} \frac{I_{\mathrm{S}}}{(I_{\mathrm{S}} + hI_{\mathrm{B}})^{1/2}} \,. \tag{1}$$

The detective quantum efficiency (DQE) for the camera used is known from [4] and amounts to 0.3, almost invariant to dose conditions. h is the statistical error describing background subtraction—in our case from the zero-loss peak flanks. For core-loss analysis and the respective cross-sections, h typically lies in the order of 5-10. In the low-loss region we expect a lower h value due to relatively higher signals. I_{B} is the background (zero-loss) intensity and I_{S} is the intensity of the signal after zero-loss removal. For our analysis, both integrals were summed over a range of 0.2 eV, the same value used for the extraction of the EELS maps in the experiment.

We next calculated the SNR for the cuboid sample for the dipolar and quadrupolar modes for projections at $0°$ tilt and at $-75°$ tilt, and conservatively estimated $h = 5$ (Supplementary Fig. 8). For this case the SNR takes on values above 10 and is well beyond the critical threshold of 3 (98% certainty of detection) for all relevant locations. We therefore conclude that the data were of sufficient statistical quality for a reliable reconstruction. An additional observation was that the SNR for electron trajectories outside the particles was even higher, which is explicable with the reduction of the signal due to concurrent scattering events. This effect is present especially at large tilt angles due to the larger effective thicknesses of the sample.

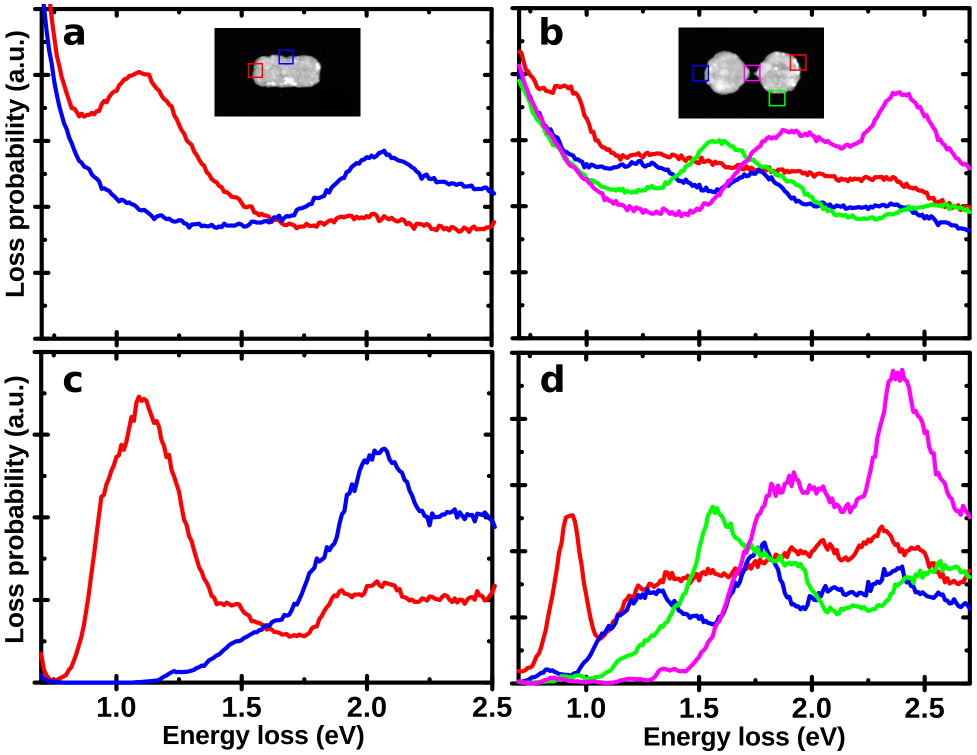

Supplementary Fig. 1: Effect of Richardson-Lucy deconvolution: **a,b** EELS spectra for cuboid and coupled disks before deconvolution, and **c,d** after deconvolution. Spectra recorded at same positions as in main text.

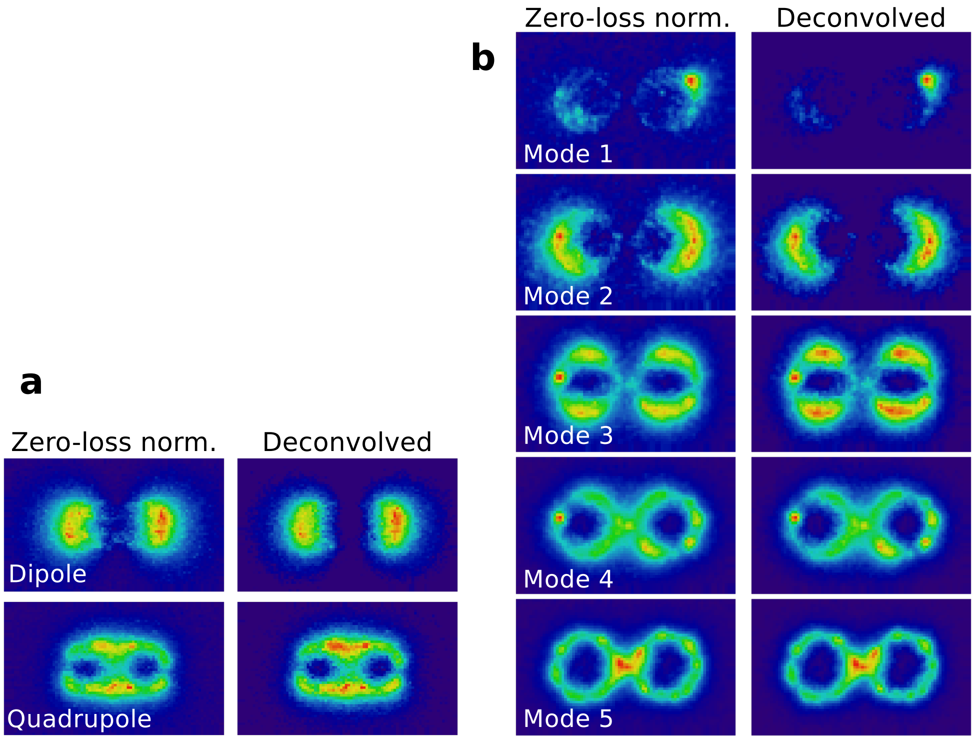

Supplementary Fig. 2: Effect of Richardson-Lucy deconvolution on EELS maps for: **a** nanocuboid and **b** coupled disks.

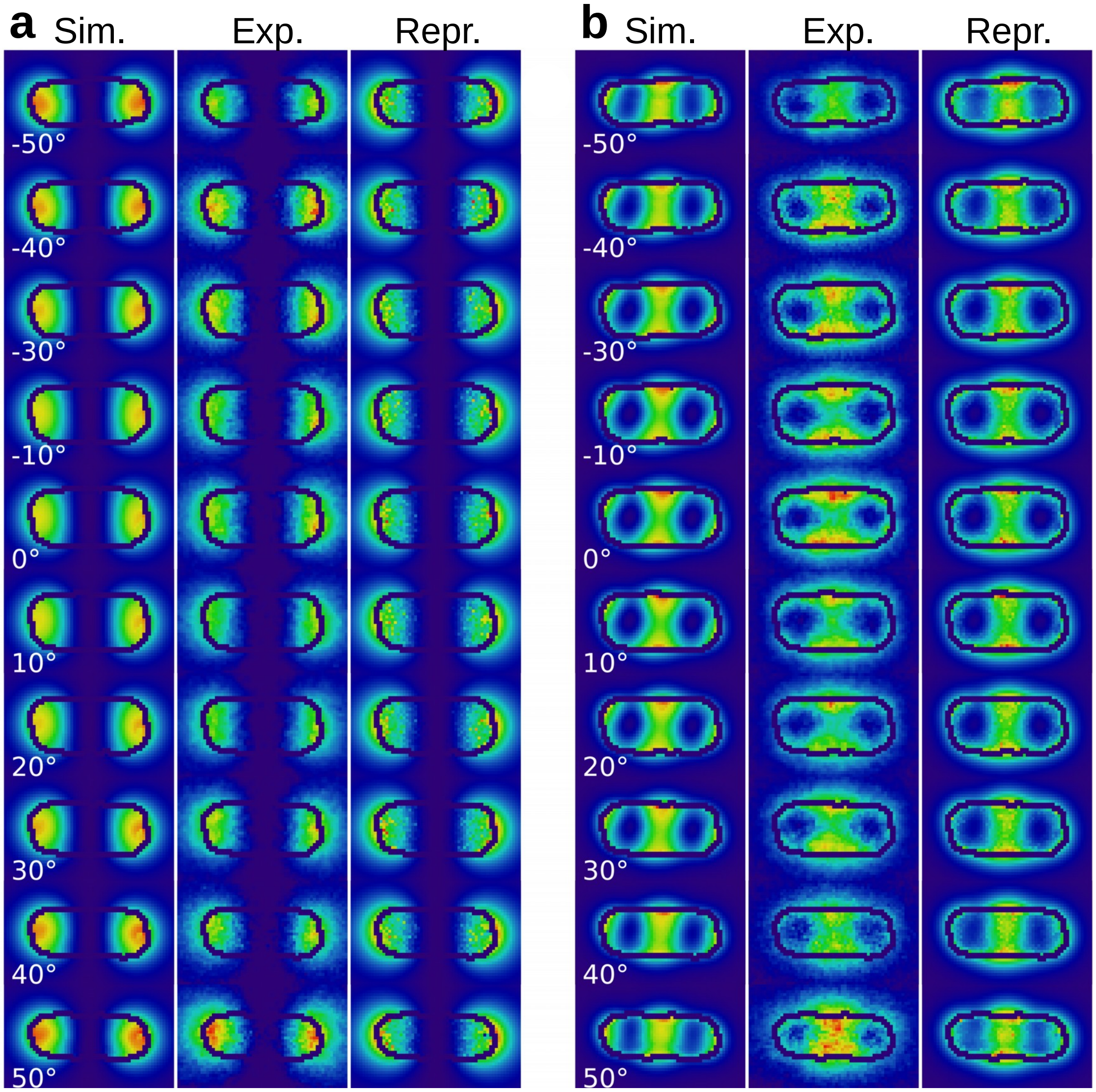

Supplementary Fig. 3: Comparison between simulated, experimental, and reprojected EELS maps for different tilt angles and for **a** dipolar mode and **b** quadrupolar mode of nanocuboide. Data from pixels at black margins around the particles were not used for the reconstruction.

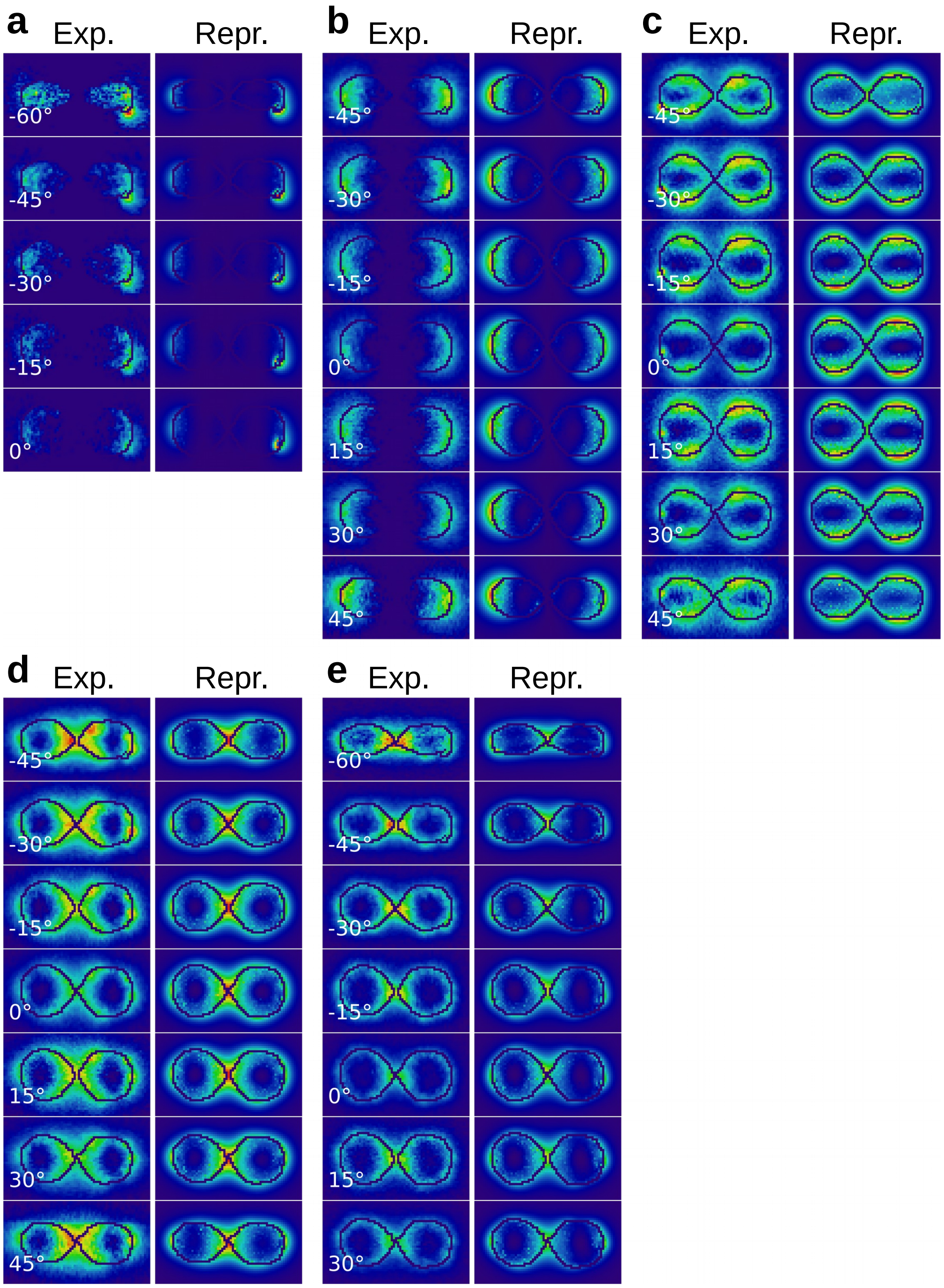

Supplementary Fig. 4: Same as Supplementary Fig. 3 but for coupled disks (without simulation results).

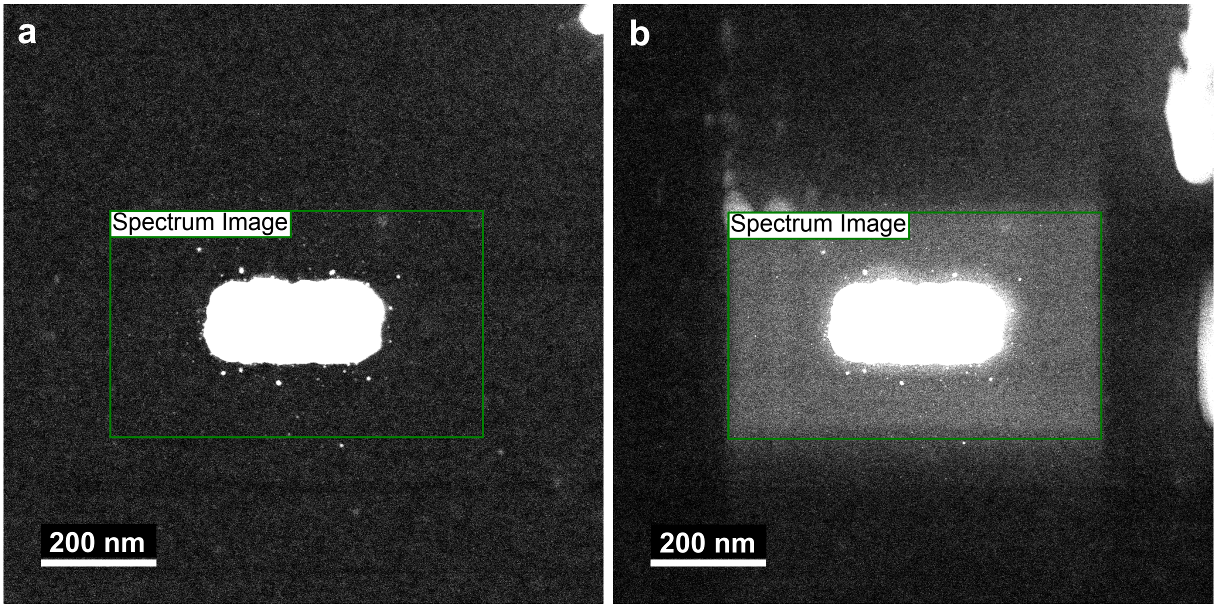

Supplementary Fig. 5: HAADF STEM images **a** before and **b** after tilt series acquisition. Contrast is adjusted to show the carbon contamination deposited during the experiment.

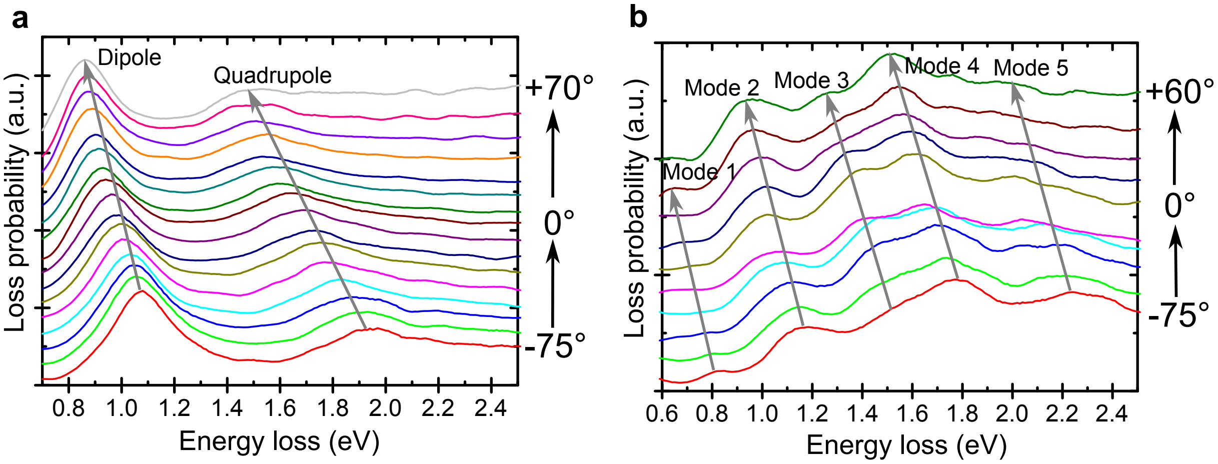

Supplementary Fig. 6: EELS spectra summed over the full spectrum image for **a** nanocuboid and **b** coupled disks. Arrows indicate shift of resonances during spectrum image acquisition for different tilt angles.

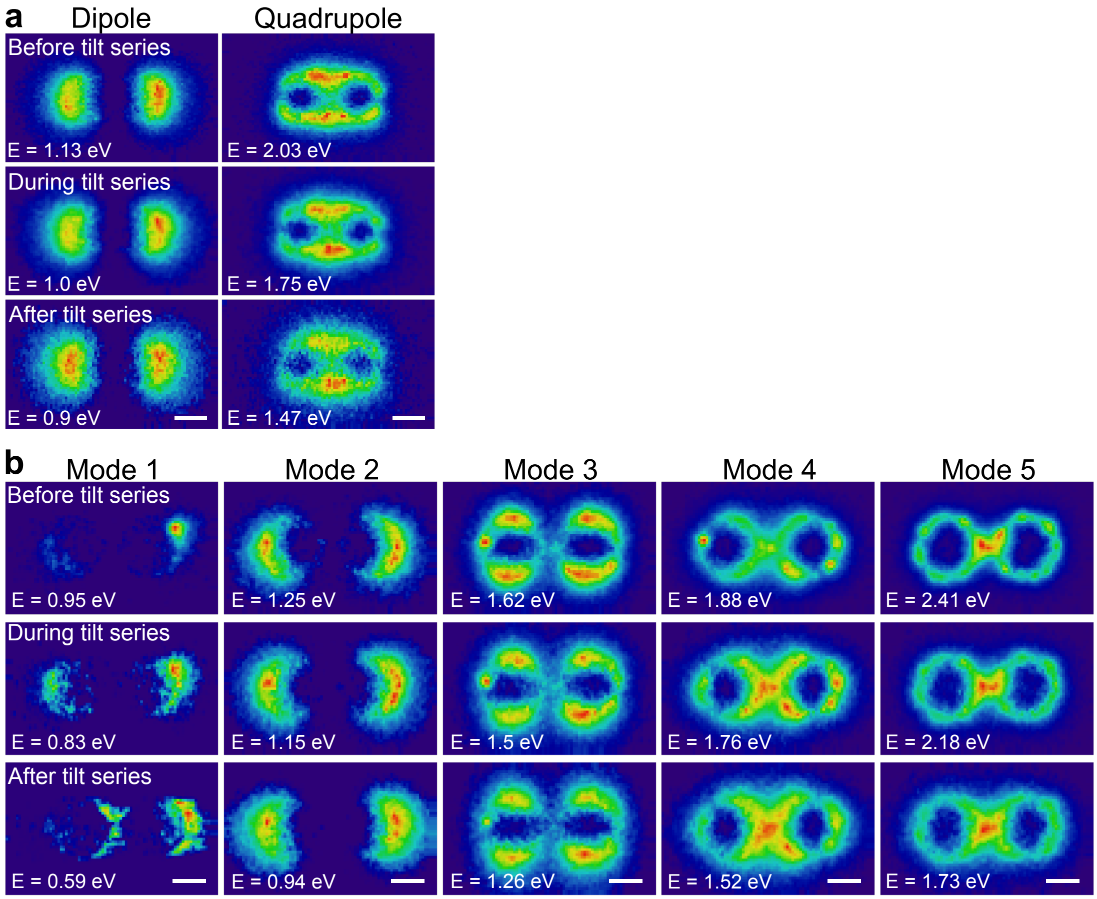

Supplementary Fig. 7: EELS maps before, during, and after tilt series at 0° tilt angle at resonance energies reported in each panel. **a** Dipolar and quadupolar mode of cuboid, and **b** modes of coupled disks. Scale bars are 100 nm.

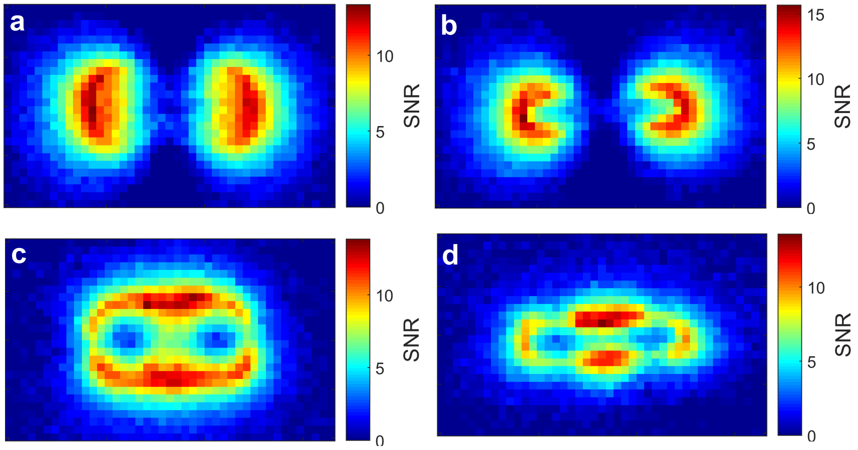

Supplementary Fig. 8: Calculated SNR maps for the dipolar mode at **a** $0°$ tilt and **b** $-75°$ tilt, and for the quadupolar mode at **c** $0°$ tilt and **d** $-75°$ tilt.

Supplementary References

[1] O. Nicoletti, F. de la Pena, R. W. Leary, D. J. Holland, C. Ducati, and P. A. Midgley. Three-dimensional imaging of localized surface plasmon resonances of metal nanoparticles. *Nature* **502**, 80–84 (2013).

[2] G. Haberfehlner, A. Trügler, F. P. Schmidt, A. Hörl, F. Hofer, U. Hohenester, and G. Kothleitner. Correlated 3d nanoscale mapping and simulation of coupled plasmonic nanoparticles. *Nano Lett.* **15**, 7726–7730 (2015).

[3] R. F. Egerton. *Electron Energy-Loss Spectroscopy in the Electron Microscope*. Springer, Berlin, 2011.

[4] K. Riegler and G. Kothleitner. EELS detection limits revisited: Ruby — a case study. *Ultramicroscopy* **110**, 1004–1013 (2010).
